# Preoperative prediction of CNS WHO grade and tumour aggressiveness in intracranial meningioma based on radiomics and structured semantics

**DOI:** 10.1038/s41598-024-71200-0

**Published:** 2024-09-04

**Authors:** Darius Kalasauskas, Michael Kosterhon, Elena Kurz, Leon Schmidt, Sebastian Altmann, Nils F. Grauhan, Clemens Sommer, Ahmed Othman, Marc A. Brockmann, Florian Ringel, Naureen Keric

**Affiliations:** 1 Department of Neurosurgery, University Medical Center, Johannes Gutenberg University, Langenbeckstr. 1, 55131 Mainz, Germany; 2 Department of Neuroradiology, University Medical Center, Johannes Gutenberg University, Langenbeckstr. 1, 55131 Mainz, Germany; 3 Institute of Neuropathology, University Medical Center, Johannes Gutenberg University, Langenbeckstr. 1, 55131 Mainz, Germany

**Keywords:** Meningioma, Radiomics, Recurrence, CNS WHO grade, Prediction, CNS cancer, Surgical oncology, Cancer imaging, CNS cancer, Neoplasm staging, Brain imaging, Magnetic resonance imaging, Disease-free survival

## Abstract

Preoperative identification of intracranial meningiomas with aggressive behaviour may help in choosing the optimal treatment strategy. Radiomics is emerging as a powerful diagnostic tool with potential applications in patient risk stratification. In this study, we aimed to compare the predictive value of conventional, semantic based and radiomic analyses to determine CNS WHO grade and early tumour relapse in intracranial meningiomas. We performed a single-centre retrospective analysis of intracranial meningiomas operated between 2007 and 2018. Recurrence within 5 years after Simpson Grade I-III resection was considered as early. Preoperative T1 CE MRI sequences were analysed conventionally by two radiologists. Additionally a semantic feature score based on systematic analysis of morphological characteristics was developed and a radiomic analysis were performed. For the radiomic model, tumour volume was extracted manually, 791 radiomic features were extracted. Eight feature selection algorithms and eight machine learning methods were used. Models were analysed using test and training datasets. In total, 226 patients were included. There were 21% CNS WHO grade 2 tumours, no CNS WHO grade 3 tumour, and 25 (11%) tumour recurrences were detected in total. In ROC analysis the best radiomic models demonstrated superior performance for determination of CNS WHO grade (AUC 0.930) and early recurrence (AUC 0.892) in comparison to the semantic feature score (AUC 0.74 and AUC 0.65) and conventional radiological analysis (AUC 0.65 and 0.54). The combination of human classifiers, semantic score and radiomic analysis did not markedly increase the model performance. Radiomic analysis is a promising tool for preoperative identification of aggressive and atypical intracranial meningiomas and could become a useful tool in the future.

## Introduction

Meningiomas are the most common primary tumours of the central nervous system and comprise approximately one-third of all intracranial neoplasms. Most are solitary and might be discovered as an incidental finding in almost 3% of cranial MRIs^1^. Meningiomas are classified according to WHO as benign, slow growing CNS WHO grade 1, more aggressive, atypical grade 2, and very rare anaplastic grade 3 tumours^2^. Atypical meningiomas tend to grow faster and more aggressive in comparison to CNS WHO grade 1 tumours and are more likely to recur after tumour resection^3^. On the other hand, not all CNS WHO grade 2 tumours recur, and some recurrences are observed even after complete resection of grade 1 tumours^4^. Several factors such as completeness of resection or follow-up time may play a role but predicting tumour recurrence remains a difficult task.

Radiomics, a method of processing imaging data according to various mathematical algorithms has become a powerful diagnostic tool in recent years. The number of publications dealing with application of radiomics for diagnosis and prognosis determination in patients with central nervous system (CNS) diseases has increased rapidly in recent years. Radiomic analysis can increase the accuracy of imaging analysis by extracting data that cannot be detected by human observers^5^. Such algorithms hold promise for characterising the tumour type and behaviour of certain tumours. For meningioma, the main goals for radiomic analysis are determination of CNS WHO grade and identification of tumours with the potential for early recurrence. In the long term, the identification of aggressive tumours on preoperative MRI may assist the surgeon in adapting the resection strategy and further management, such as the application of adjuvant therapy or the frequency of follow-up imaging.

In this study, we aimed to develop a predictive model to determine CNS WHO grade and aggressive tumour behaviour in intracranial meningiomas based on radiomic and semantic features.

## Methods

We performed a single-centre retrospective analysis of patients who underwent surgery for intracranial meningiomas at our department between 2007 and 2018. The inclusion criteria for the study were: histological confirmation of intracranial meningioma with CNS WHO grade 1 or 2 (WHO grade 3 meningiomas were excluded, as there were too few cases in the database), presence of histological reports, at least one postoperative follow-up ≥ 3 months after surgery to determine aggressive behaviour. Cases with incomplete tumour resection (Simpson IV–V) likewise were excluded from further analysis. For analysis of early relapse only patients who have developed a relapse within 5 years were included. Patients with fewer than 5 years follow up without a relapse were excluded from this comparison, as they might have developed a relapse after and fall into a different cohort.

In addition to standard histopathological work-up for meningioma (Haematoxylin and eosin (HE), reticulin stain, Ki67-immunohistochemistry), telomerase reverse transcriptase (TERT) promoter mutations were assessed if available. CNS WHO grade was obtained from the histopathology report and systematically analysed.

**Fig. 1:**
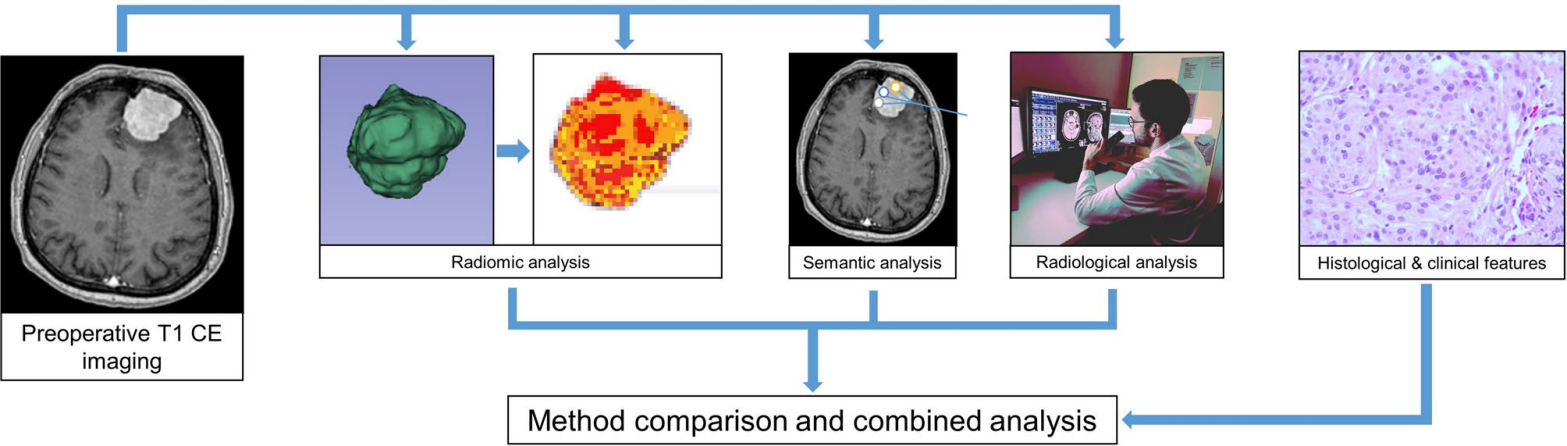
The systematic approach to imaging analysis in the study.

Beside the histopathological analysis, conventional radiological analysis, a systematic feature score analysis and a radiomics analysis were performed and evaluated as depicted in the workflow chart in Fig. 1.

### Conventional radiological analysis

Two board-certified neuroradiologists (5 and 7 years of experience, respectively) were asked to independently evaluate MRI scans regarding tumour aggressiveness and WHO grade while being blinded to the actual WHO grade and clinical course. Provided with contrast-enhanced (CE) T1 sequences only, they were asked to classify a meningioma to be aggressive or not. Aggressiveness here was defined as tumours with a propensity to develop a recurrence within 5 years. Furthermore, Neuroradiologists were asked to give a guess about the tumour to be more likely WHO grade 1 or 2.

### Systematic morphological analysis with a semantic feature score

In addition, a systematic analysis of typical semantics or terms often used in radiological reports to describe the morphological aspect of a tumour was performed. All MRIs were screened and lesions were labeled with the appropriate morphological features depicting aggressive behaviour^6,7^. A total of 11 different items were assessed: “intratumoural heterogeneity”, “multifocality”, “midline shift”, “sinus invasion”, “necrosis/haemorrhage”, “mass effect”, “cystic component”, “hyperostosis”, “bone invasion”, “spiculation” and “edema”. To evaluate the association between these atypical features and aggressive clinical course the number of aggressive morphological features per tumour was quantified into a score and used for further analysis. This semantic score was calculated by adding 1 point for every positive feature, so that meningiomas without aggressive features would score 0 points to a maximum score of 11 points when all above mentioned aggressive features were present.

### Radiomic analysis

Radiomic analysis was performed on preoperative MRI scan with CE T1 sequences. In case of insufficient quality (motion artefacts, distortions, inability to import into the software etc.), the acquisition was not used for radiomic feature extraction. In patients with multiple MRI scans, the most recent scan prior to resection was selected. The source of MRI (Department of Neuroradiology or other sources) slice thickness (thin (1 mm) vs. thick (3–5 mm)-sliced MRI) were recorded and used for further analysis.

Tumour volume was manually extracted from contrast-enhanced MRI T1 sequences using 3D Slicer software (www.slicer.org) by manual segmentation^8,9^ to generate the region of interest (ROI) for further radiomic analysis. Radiomic analysis was performed using the PyRadiomics add-on for 3DSlicer^8^. During pre-processing, voxel intensity was normalized to a scale of 0 to 100, pixel spacing was resampled as needed to 1 × 1 × 1 mm, bin width was set to 25 and symmetric Gray Level Co-occurrence Matrix (GLCM) was used. The following radiomic classes were used: First order statistics, Gray Level Dependence Matrix (GLDM), Shape descriptcaors, GLCM, Gray Level Run Length Matrix (GLRLM), Gray Level Size Zone Matrix (GLSZM), Neighboring Gray Tone Difference Matrix (NGTDM) and filtered features. For filtering, a wavelet filter was used in combination with the features. This resulted in a total of 791 radiomic features. The datasets were normalized using *z*-scores. One of eight selection algorithms (Information gain, Information gain ratio, Gini-Decrease, ANOVA, Chi Square, LASSO, ReliefF, Fast Correlation Based Filter (FCBF)) was applied to select the 25 most informative radiomic features for the further model building. Eight machine learning approaches (Random Forest, AdaBoost, Gradient boosting, Naïve Bayes, Support Vector Machines (SVM), Neural Networks, Logistic regression and k-Nearest Neighbour) were applied for model building using Orange software (www.orangedatamining.com, University of Ljubljana, Slovenia) to determine WHO grade and early tumour recurrence. The data was divided into 66% training and 34% test data. The split was stratified to ensure each subset contains the same percentage of e.g. CNS WHO 1 and 2 cases (79% vs. 21%). Different combinations of feature selection methods and classifier algorithms were evaluated by the area under the curve (AUC) of the receiver operating characteristics (ROC) analysis’ curve, classification accuracy (CA), F1-score and Matthews correlation coefficient (MCC) to find the best performing model. The workflow of the study is depicted in Fig. 1.

The radiologists’ performance was compared to the radiomic model. The model was improved by adding certain semantic and histopathological features. *p* < 0.05 was considered to be significant.

## Results

### Clinical findings

A total of 226 patients (76% women, mean age 58.6 (SD 13.4) years) were included in the study. There were 79% WHO grade 1 and 21% WHO grade 2 tumours (Table 1). Overall, 25.7% of tumours demonstrated certain putative aggressive histopathological features, such as high mitotic count, high Ki-67 index or necrosis.

**Table 1:** Patient and tumour characteristics.

Characteristics	
Number of patients, total	226
Female, (%)	171 (75.7)
Age, mean (SD)	58.6 (13.4)
CNS WHO tumour grade	
Number of grade 1, (%)	178 (78.8)
Number of grade 2, (%)	48 (21.2)
Follow-up, months, mean (SD)	54.0 (42.0)
Progression-free survival, months, mean (SD)	123.4 (5.0)
Tumour recurrence, (%)	25 (11.1)
Number of tumour recurrences within 5 years, total (%)	19 (18.4)
Recurrence in grade 1, (%)	11 (20.0)
Recurrence in grade 2, (%)	8 (32.0)

We found 25 (11%) tumour recurrences, and the mean progression-free survival (PFS) was 123.4 (SE 5.3) months. 19 recurrences occurred within 5 years after operation, while 56 patients had a follow up longer than 5 years and experienced no tumour relapse. There were 11 (20.0%) recurrences in CNS WHO grade 1 and 8 (32.0%) recurrences in CNS WHO grade 2 meningiomas within 5 years (*p* = 0.26), the high number of tumour recurrences might be associated with certain selection bias. The presence of irregular histopathological features was not associated with tumour relapse (*p* = 0.26).

The surgical reports were screened for the Simpson grade of resection completeness. In CNS WHO grade 1 meningeomas, Simpson degrees as follows were achieved: grade I (n = 76, 42.7%), grade II (n = 73, 41.0%), grade III (n = 29, 16.3%). For WHO grade 2 tumours, Simpson grades I (n = 33, 68.8%), grades II (n = 11, 22.9%), grades III (n = 4, 8.3%) were documented. No association between Simpson grade (I to III) and tumour relapse within 5 years was found (*p* = 0.168).

### Results of radiological assessment

**Fig. 2:**
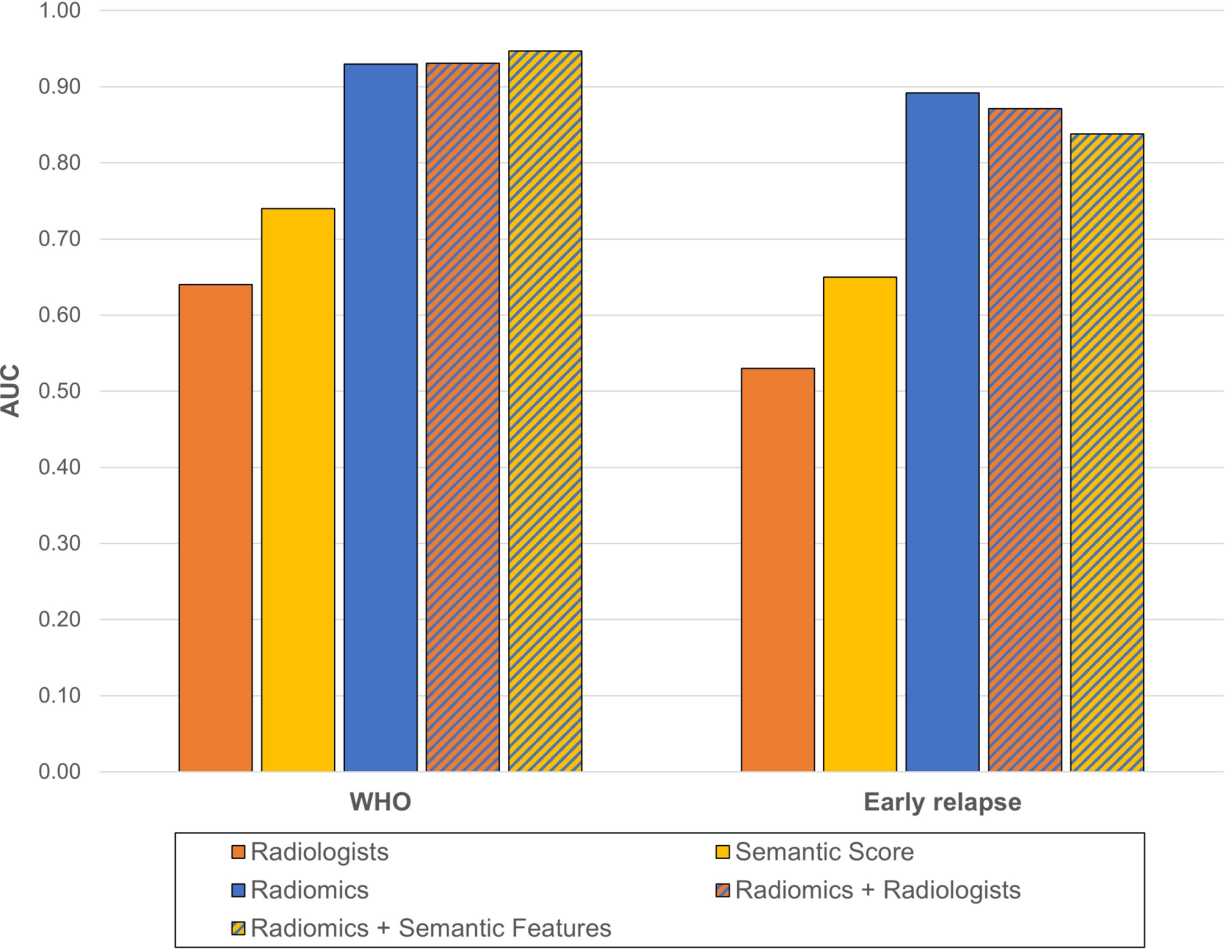
Model performance for determination of CNS WHO grade and early recurrence in intracranial meningioma.

The two neuroradiologists classified 65.9% and 60.6% of meningiomas as CNS WHO grade 1 and 24.6% and 30.1% as CNS WHO grade 2 (Intraclass correlation coefficient (ICC) 0.93 (95% confidence interval (95% CI) 0.90–0.95)). Aggressive features were classified in 7.1% and 8.8% of tumours (ICC 0.94, 95%CI 0.92–0.95). The AUC for determination of CNS WHO grade by radiologists compared to histological CNS WHO grade was 0.64 and 0.65 (CA 68.7% and 72.7%) and for determination of aggressive meningioma behaviour in terms of clinical recurrence 0.53 and 0.54 (CA 71.8% and 73.2%, respectively) (Fig. 2).

### Semantic feature score

**Fig. 3:**
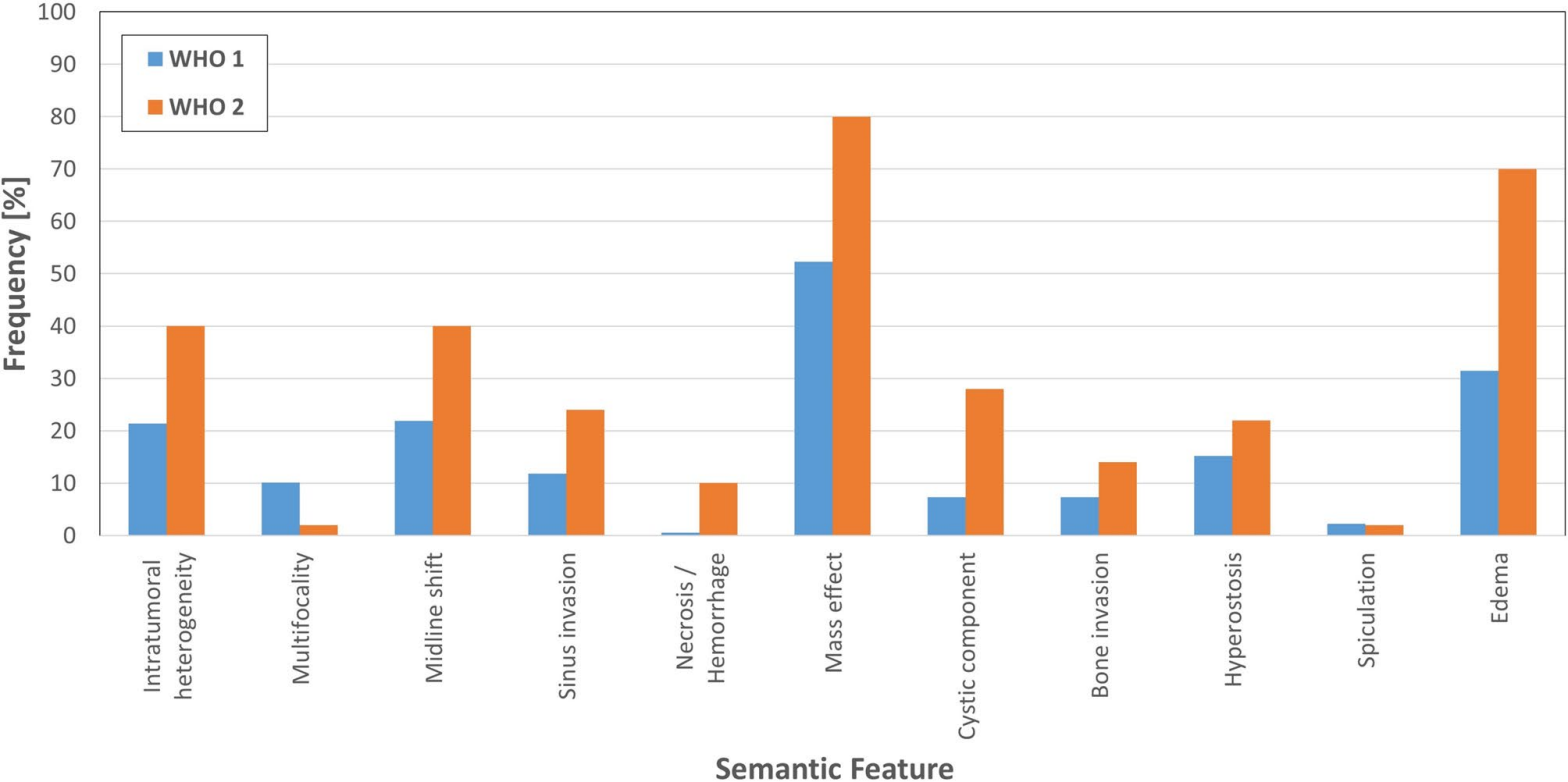
Distribution of morphological features in CNS WHO grade 1 and 2 meningiomas.

**Fig. 4:**
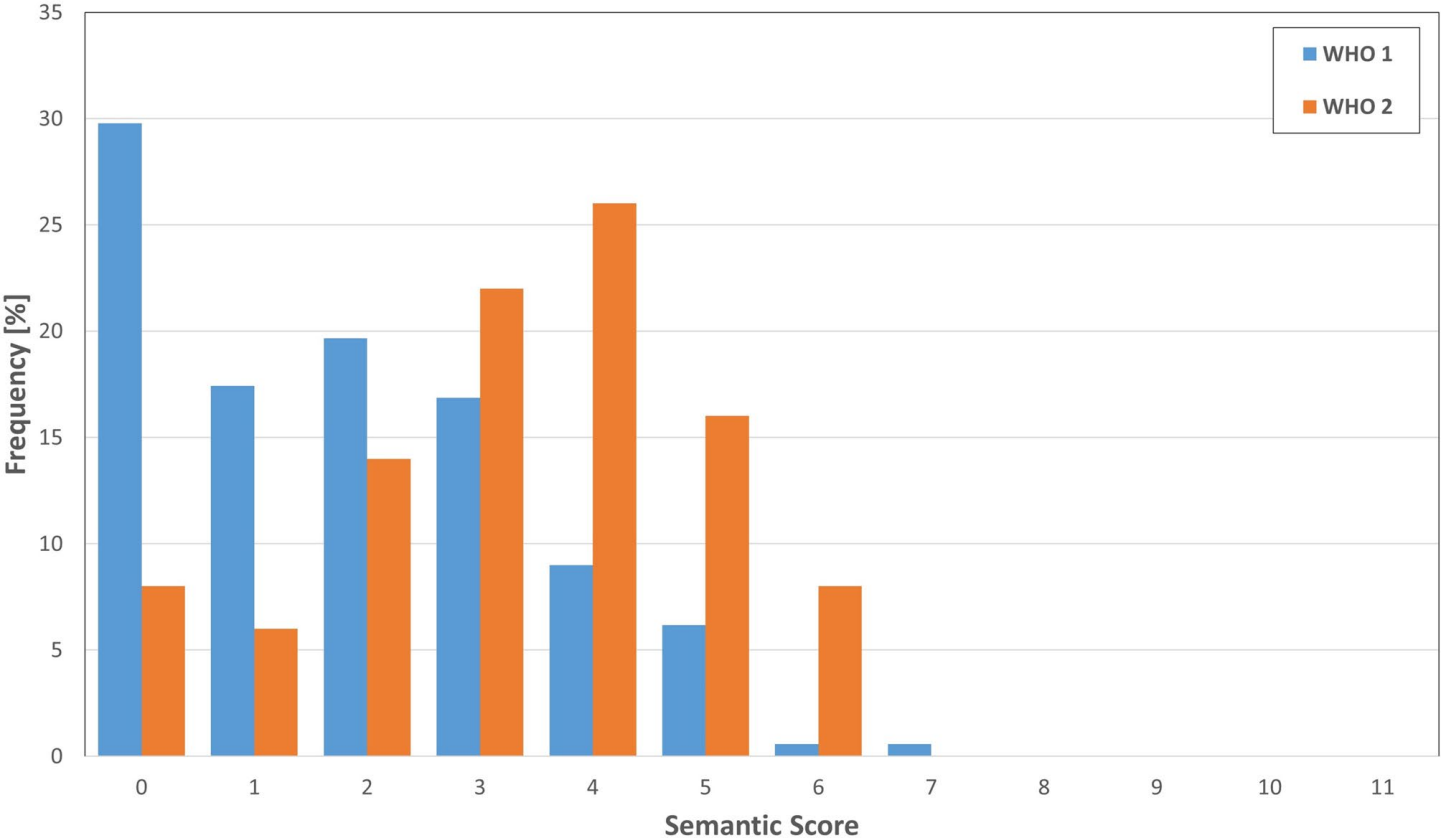
Distribution of the Semantic Score in CNS WHO grade 1 and 2 meningiomas.

Analysis of morphologic aggressive features showed their abscence in only 22% of cases (semantic feature score = 0). The most common aggressive features were “mass effect” (50.7%) and “edema” (34.7%) (Fig. 3). The semantic score demonstrated quantitatively more aggressive features in CNS WHO grade 2 tumours (Fig. 4) and the score was associated with the radiologist assessment of aggressive tumours (AUC 0.84 and 0.79), and CNS WHO grade 2 tumours (AUC 0.85 for both). The AUC for determination of tumour recurrence within 5 years was slightly better than radiological assessment (AUC 0.65) (Fig. 2). However, the score performed better in prediction of CNS WHO grade 2 histopathology (AUC 0.74) (Fig. 2), optimal cut-off according to Youden’s index was 3 points (Fig. 3). Furthermore logistic regression was performed and a nomogram was created to compare the features regarding their influence by the means of their log odds ratios for predicting CNS WHO grade 2 and tumour relapse. The top 3 features in terms of CNS WHO grade 2 prediction were “Necrosis/Hemorrhage” (LogOR = 2.21), “Edema” (LogOR = 1.32) and “Cystic component” (LogOR = 1.06). In terms of early relapse the most influential features were “Bone invasion” (LogOR = 1.49), “Cystic component” (LogOR = 1.32) and “Spiculation” (LogOR = 1.04).

### Radiomic prediction model

After extracting the 791 radiomic features for each tumour, we tested 8 different algorithms to select the most relevant features (Information gain, Information gain ratio, Gini-Decrease, ANOVA, Chi Square, LASSO, ReliefF, Fast Correlation Based Filter (FCBF)). Features were ranked according to their contribution to the target variable for each selection method. The whole dataset with 262 patients was split into 173 patients (66%) for the training set and 89 (34%) for the test set. The split was performed with stratified sampling to obtain the same percentage of e.g. CNS WHO grade 2 tumours in each group. Then 8 different machine learning classifiers were used to build the model and to be tested in the test set (AdaBoost, Gradient Boosting, k-nearest-neighbours (kNN), Logistic Regression, Naive Bayes, Neural Network, Random Forest and Support vector machines (SVM)).

We tested different combinations of feature selection method, number of best ranked features and machine learning classifier and compared measures such as AUC under the ROC, classification accuracy (CA), the F1-score and the MCC to find the best combination. For practicability and to reduce the total amount of features, only a fixed number of the best ranked 10, 25, 50 and 100 features for each selection method and each machine learning classifier were used.

For prediction of CNS WHO grade the best combination was found for LASSO as feature selection method with 25 features and a neuronal network as machine learning classifier (1000 neurons in one hidden layer, activation function: tanh, Solver: Adam). This combination not only yielded the highest AUC of 0.930 (CA: 86.5%, 95% CI ± 7.10%) but also the best MCC (0.670) which is known to provide better interpretation especially for unbalanced datasets (Supplementary Fig. 1, left table).

**Fig. 5:**
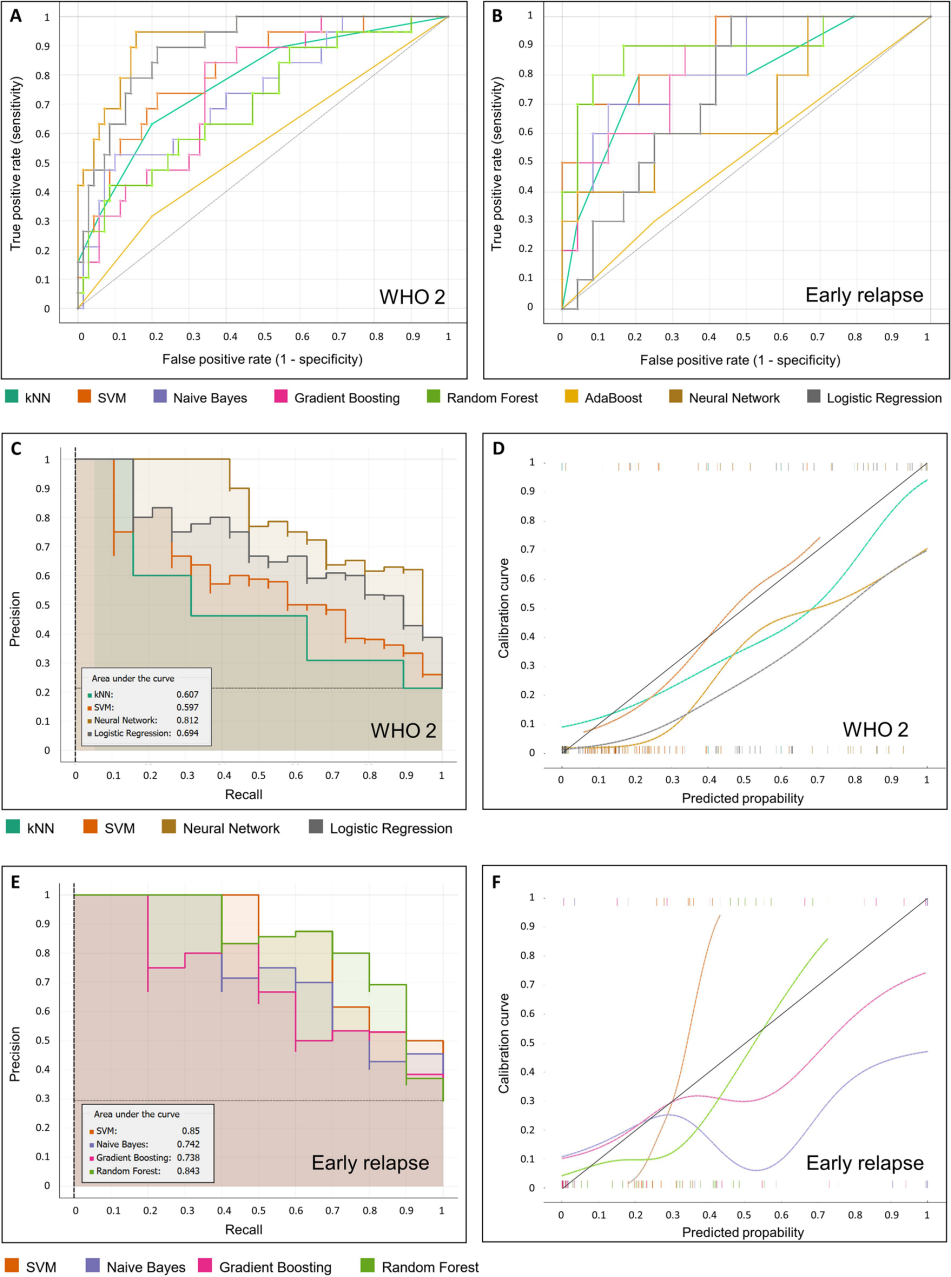
Performance metrics of different machine learning classifiers to detect CNS WHO grade 2 and early recurrence: ROC curves for detecting CNS WHO grade 2 (all with LASSO as feature selection method) (**A**), ROC curves for detecting early recurrence (all with Information Gain as feature selection method) (**B**), Precision–Recall curve (**C**) and calibration plot (**D**) for the 4 best performing classifiers for detecting CNS WHO grade 2 (all with LASSO as feature selection method), Precision–Recall curve (**E**) and calibration plot (**F**) for the 4 best performing classifiers for detecting early relapse (all with Information Gain as feature selection method).

In the prediction of early tumour recurrence the total amount of patients was 102 (lower due to partially lost follow ups) which were split in 68 for training and 34 for testing. The best combination regarding only the AUC was Information gain for feature selection with 25 features and support vector machines as classifier with a value of 0.896. However this combination only yielded a F1-Score of 0.182 and a MCC of 0.270. Therefore the combination of Information Gain (25 features) and Random Forest with a slightly lower AUC of 0.892 but much higher CA of 88.2% (95% CI ± 10.84%), F1-Score (0.750) and MCC (0.717) was chosen for further evaluation (Random Forest was performed with 50 trees, replicable training and balanced class distributions) (Supplementary Fig. 1, right table). The AUC under the ROC curve, CA, F1 score and MCC for all 8 models in combination with the best feature selection method is plotted in Fig. 5A,B.

In many clinical studies there is posed a significant interest on the minority class in the categorization, as it holds true also in this study (WHO grade II, early relapse). We therefore also analyzed the precision-recall-curve for the best 4 models in each category (Fig. 5C,E). This demonstrated an AUC under the precision-recall curve of 0.812 for Neural Networks in detection of CNS WHO grade 2 and 0.843 for Random Forest in detection of early relapse. Moreover we analysed the calibration plots for the best 4 models in each category (Fig. 5D,F).

The best models in each category were also combined with human classifiers (radiological analysis) and semantic analysis and evaluated in the respective test sets. The combination of radiologists opinion to the radiomics model did not markedly increase the models performance to predict CNS WHO grade 2 (AUC 0.931, CA 88.8% (95% CI ± 6.55%)) and it even decreased for predicting early recurrence (AUC 0.871, CA 79.4% (95% CI ± 13.59%)) (Fig. 2).

The addition of the 11 semantic items to the 791 radiomic features did slightly increase the performance of the radiomic prediction models for detecting CNS WHO grade 2 (AUC 0.947, CA 84.3% (95% CI ± 7.56%)) but decreased performance for detection of early recurrence (AUC 0.838, CA 79.4% (95% CI ± 13.59%) ) (Fig. 2).

The same was found when using the semantic score in combination with the radiomics model for CNS WHO grade (AUC 0.936, CA 85.4% (95% CI ± 7.34%)) and for early recurrence (AUC 0.829, CA 76.5% (95% CI ± 14.25%)).

### Effect of different MRI scanners

In many cases, radiomic analysis is performed on relatively homogenous data, in order to increase the predictive accuracy. This may limit the practicality of the approach, as many patients have MRIs that were performed with different devices and in various settings and centres. In some cases the initial MRIs analysed in this study were obtained from other centers but the patients were treated and followed in our hospital. Therefore, to investigate the impact of imaging heterogeneity in our study, we performed an additional analysis excluding all MRI scans performed in centres other than ours. Inclusion of MRI scans from only one centre (N = 200 for CNS WHO grade and N = 78 for early relapse) showed a further improvement of the models performance with an AUC of 0.966 (CA 92.6%, 95% CI ± 6.22%) for detection of CNS WHO grade but no improvement of model performance for early recurrence with an AUC of 0.868 (CA 80.8%, 95% CI ± 15.14%) was found.

## Discussion

This study demonstrates that the radiomic analysis can be applied to determine tumour aggressiveness and CNS WHO grade. This approach was superior in comparison to experienced neuroradiologists and morphological analysis in terms of the proposed semantic feature score. Moreover, it suggests that MRIs from heterogeneous sources can be used for this purpose after appropriate pre-processing.

There are several established risk factors for meningioma recurrence, such as WHO grade, genetic markers, methylation profile or brain invasion^2^, as well as Simpson grade^10^, whereas the definition of complete resection remains debatable^11^. Other factors have been identified such as patient age, performance score, tumour size, location, appearance or morphology throughout numerous retrospective studies^4,12–14^ were suggested. Apparently, in many cases the tendency of recurrence of scull base meningiomas depends on surgical reasons, as often a complete resection is not possible due to infiltration of adjacent relevant structures^15,16^. Therefore, we excluded the cases with incomplete tumour resection from further work-up.

As some previous studies have shown, the radiomic approach can be helpful in the preoperative workup of intracranial meningiomas: it can be used for differentiation between meningiomas and solitary fibrous tumours^17,18^ or tumours in the sellar region (pituitary adenoma, craniopharyngioma, meningioma)^19,20^. Some authors have also addressed the differentiation of histologic tumour subtype^21^, tumour aggressiveness^22,23^ or CNS WHO grade^24,25^ with considerable accuracy. In addition, some authors analysed/predicted tumour consistency^26,27^ or bone invasion^28^, which may be a considerable benefit for surgical planning. Brain invasion could also be predicted in some studies ^29–32^, although its prognostic significance remains a matter of discussion^33,34^.

In our study the use of semantic features or semantic scores have demonstrated an AUC above 0.74 for tumour grade and lower power for tumour aggressiveness. These tumour markers have previously been explored as predictors for tumour aggressiveness in certain cases^6,7,35^ showing that a rapid systematic morphological analysis using limited resources may provide additional information. Moreover, Morin et al. integrated radiologic, radiomic, and clinical models to establish a model for CNS WHO grade and tumour survival and achieved AUC up to 0.78, which was slightly lower than in our model^23^. In contrast, our model did not significantly increase its accuracy after semantic or radiologic features were added.

One major problem in machine learning is over fitting, meaning prediction accuracy of a model can decrease if it is fed with new data from other sources than it was originally trained on. Therefore, we tested our model with both heterogenous and relatively homogenous MRI sets, using both MRIs performed at our centre and externally. It is known, that heterogeneity of MRI data can reduce the model quality^36^, however, in our cohort, the model performed well with both heterogenous and homogenous MRI data after the data were appropriately pre-processed. Pre-processing is an important step before a radiomic analysis, as it allows normalization of radiomic features and reduces heterogeneity^37^. Nevertheless, the radiomic approach resulted in significantly higher predictive accuracy for both CNS WHO grade and tumour recurrence than radiologic assessment or the semantic score. This indicates that integration of certain aspects of such analysis into daily routine may be promising with regard to preoperative planning. It could be argued that the preoperative determination of the CNS WHO grade does not provide any additional information in patients undergoing surgery, as a histopathological examination would be performed anyway. Moreover, some of CNS WHO grade 2 meningiomas do not relapse at all, but tendentially as a group are associated with more relapses in comparison to grade 1 tumours. We therefore chose the CNS WHO grading as one of the criteria for tumour aggressiveness. Preoperative determination of aggressiveness based on diagnostic imaging would provide an attractive opportunity to adjust the treatment strategy, for example opting for “watch and wait” in the case of non-aggressive meningiomas or for aggressive resection followed by adjuvant radiotherapy in the case of aggressive tumours. This might also have impact on the surgical procedure, e.g. when resecting a falcine meningioma, it might encourage the surgeon to take extra time and resect the dural attachment to achieve Simpson Grade 1 resection instead of coagulating the dural attachment only.

Besides, integration of radiologic, radiomic and histopahtological factors could help to identify patients most likely to experience early tumour relapse. This might help e.g. to stratify patients according to their risk of tumour recurrence into groups and to obtain more or less frequent follow-up MRI scans.

## Limitations

This study has several limitations. First, it is a retrospective study of a single-centre, without an external validation set. Therefore, the results should be verified with an external cohort. In some cases, only limited histopathologic or clinical data were available. Numerous radiomic features as predictors in small samples may lead to over fitting and false-positive findings^38,39^. Methods for dimensionality reduction, such as random projection algorithms, feature selection using unsupervised methods such as principle component analysis may reduce model overfitting^40^. Although our dataset had a relatively large number of cases in total the number of CNS WHO grade 2 cases (n = 48) and especially tumour recurrences (n = 19) was low. Splitting data into train and test set is crucial in machine learning to avoid overfitting. However in small datasets this may lead to even smaller test sets. Recognizing this as a limitation, we decided as a trade-off to overcome this issue to perform feature selection first in the whole cohort and then split the data for training the classifiers. Feature selection was done using logistic regression with L1 (LASSO) regularization to at least reduce overfitting issues here.

As mentioned the number of CNS WHO grade 3 meningeomas is known to be rare but their detection in advance solely from radiomic markers would be particularly important for further clinical management. Due to the small number in our data set, these were not evaluated and synthetic data augmentation approaches (e.g. SMOTE approach) were deliberately avoided. Their reliable detection requires multi-center data with more patients.

## Conclusions

Radiomic analysis is a promising tool for preoperative identification of aggressive and atypical intracranial meningiomas and could become a useful tool in the future for therapeutic strategies, clinical decision making, and patient counselling.

## Supplementary Information

Supplementary Figure S1.

## Data Availability

Parts of the dataset used and analysed during the current study are available in a de-identified version from the corresponding author on reasonable request.

## References

[1] Vernooij, M. W. *et al.* Incidental findings on brain MRI in the general population. *N .Engl. J. Med.* **357**, 1821–1828. 10.1056/NEJMoa070972 (2007). 17978290 10.1056/NEJMoa070972

[2] Louis, D. N. *et al.* The 2021 WHO classification of tumours of the central nervous system: A summary. *Neuro Oncol.* **23**, 1231–1251. 10.1093/neuonc/noab106 (2021). 34185076 10.1093/neuonc/noab106 PMC8328013

[3] Champeaux, C., Wilson, E., Shieff, C., Khan, A. A. & Thorne, L. WHO grade II meningioma: A retrospective study for outcome and prognostic factor assessment. *J. Neurooncol.* **129**, 337–345. 10.1007/s11060-016-2181-2 (2016). 27311726 10.1007/s11060-016-2181-2

[4] Corniola, M. V. & Meling, T. R. Management of recurrent meningiomas: State of the art and perspectives. *Cancers (Basel).*10.3390/cancers14163995 (2022). 36010988 10.3390/cancers14163995 PMC9406695

[5] Yi, Z., Long, L., Zeng, Y. & Liu, Z. Current advances and challenges in radiomics of brain tumours. *Front. Oncol.* **11**, 732196. 10.3389/fonc.2021.732196 (2021). 34722274 10.3389/fonc.2021.732196 PMC8551958

[6] Kalasauskas, D. *et al.* Identification of high-risk atypical meningiomas according to semantic and radiomic features. *Cancers (Basel).*10.3390/cancers12102942 (2020). 33053798 10.3390/cancers12102942 PMC7599676

[7] Coroller, T. P. *et al.* Radiographic prediction of meningioma grade by semantic and radiomic features. *PLoS One* **12**, e0187908. 10.1371/journal.pone.0187908 (2017). 29145421 10.1371/journal.pone.0187908 PMC5690632

[8] van Griethuysen, J. J. M. *et al.* Computational radiomics system to decode the radiographic phenotype. *Cancer Res.* **77**, e104–e107. 10.1158/0008-5472.CAN-17-0339 (2017). 29092951 10.1158/0008-5472.CAN-17-0339 PMC5672828

[9] Kikinis, R., Pieper, S. D. & Vosburgh, K. G. in *Intraoperative Imaging and Image-Guided Therapy* (ed F. A. Jolesz) 277–289 (Springer, 2014).

[10] Simon, M. & Gousias, K. Grading meningioma resections: The Simpson classification and beyond. *Acta Neurochirurgica* **166**, 28. 10.1007/s00701-024-05910-9 (2024). 38261164 10.1007/s00701-024-05910-9 PMC10806026

[11] Behling, F. *et al.* The role of Simpson grading in meningiomas after integration of the updated WHO classification and adjuvant radiotherapy. *Neurosurg. Rev.* **44**, 2329–2336. 10.1007/s10143-020-01428-7 (2021). 33104905 10.1007/s10143-020-01428-7 PMC8338836

[12] Corniola, M. V. *et al.* Posterior fossa meningiomas: Perioperative predictors of extent of resection, overall survival and progression-free survival. *Acta Neurochirurgica* **161**, 1003–1011. 10.1007/s00701-019-03862-z (2019). 30859321 10.1007/s00701-019-03862-z

[13] Ren, L. *et al.* The development of a combined clinico-radiomics model for predicting post-operative recurrence in atypical meningiomas: A multicenter study. *J. Neurooncol.* **166**, 59–71. 10.1007/s11060-023-04511-3 (2024). 38146046 10.1007/s11060-023-04511-3

[14] Domingues, P. H. *et al.* Proposal for a new risk stratification classification for meningioma based on patient age, WHO tumour grade, size, localization, and karyotype. *Neuro Oncol.* **16**, 735–747. 10.1093/neuonc/not325 (2014). 24536048 10.1093/neuonc/not325 PMC3984558

[15] Ketter, R. *et al.* Correspondence of tumour localization with tumour recurrence and cytogenetic progression in meningiomas. *Neurosurgery* **62**, 61–69. 10.1227/01.neu.0000311062.72626.d6 (2008) (**discussion 69–70**). 18300892 10.1227/01.neu.0000311062.72626.d6

[16] Splavski, B., Hadzic, E., Bagic, I., Vrtaric, V. & Splavski, B. Jr. Simple Tumour localization scale for estimating management outcome of intracranial meningioma. *World Neurosurg.* **104**, 876–882. 10.1016/j.wneu.2017.05.039 (2017). 28526644 10.1016/j.wneu.2017.05.039

[17] Wei, J. *et al.* Accurate preoperative distinction of intracranial hemangiopericytoma from meningioma using a multihabitat and multisequence-based radiomics diagnostic technique. *Front. Oncol.* **10**, 534. 10.3389/fonc.2020.00534 (2020). 32509567 10.3389/fonc.2020.00534 PMC7248296

[18] Dong, J. *et al.* Differential diagnosis of solitary fibrous tumour/hemangiopericytoma and angiomatous meningioma using three-dimensional magnetic resonance imaging texture feature model. *Biomed. Res. Int.* **2020**, 5042356. 10.1155/2020/5042356 (2020). 33344637 10.1155/2020/5042356 PMC7725548

[19] Zhang, Y. *et al.* Machine-learning classifiers in discrimination of lesions located in the anterior skull base. *Front. Oncol.* **10**, 752. 10.3389/fonc.2020.00752 (2020). 32547944 10.3389/fonc.2020.00752 PMC7270197

[20] Zhang, Y., Chen, C., Tian, Z., Cheng, Y. & Xu, J. Differentiation of pituitary adenoma from Rathke cleft cyst: Combining MR image features with texture features. *Contrast Media Mol. Imaging* **2019**, 6584636. 10.1155/2019/6584636 (2019). 31741657 10.1155/2019/6584636 PMC6854938

[21] Niu, L. *et al.* Differentiation researches on the meningioma subtypes by radiomics from contrast-enhanced magnetic resonance imaging: A preliminary study. *World Neurosurg.* **126**, e646–e652. 10.1016/j.wneu.2019.02.109 (2019). 30831287 10.1016/j.wneu.2019.02.109

[22] Zhang, Y. *et al.* Radiomics approach for prediction of recurrence in skull base meningiomas. *Neuroradiology* **61**, 1355–1364. 10.1007/s00234-019-02259-0 (2019). 31324948 10.1007/s00234-019-02259-0 PMC7717070

[23] Morin, O. *et al.* Integrated models incorporating radiologic and radiomic features predict meningioma grade, local failure, and overall survival. *Neurooncol. Adv.* **1**, vdz011. 10.1093/noajnl/vdz011 (2019). 31608329 10.1093/noajnl/vdz011 PMC6777505

[24] Kim, S. *et al.* Comparison of diagnostic performance of two-dimensional and three-dimensional fractal dimension and lacunarity analyses for predicting the meningioma grade. *Brain Tumour Res. Treat* **8**, 36–42. 10.14791/btrt.2020.8.e3 (2020). 10.14791/btrt.2020.8.e3 PMC7221468 32390352

[25] Zhu, Y. *et al.* A deep learning radiomics model for preoperative grading in meningioma. *Eur. J. Radiol.* **116**, 128–134. 10.1016/j.ejrad.2019.04.022 (2019). 31153553 10.1016/j.ejrad.2019.04.022

[26] Cepeda, S. *et al.* Meningioma consistency can be defined by combining the radiomic features of magnetic resonance imaging and ultrasound elastography. A pilot study using machine learning classifiers. *World Neurosurg.* **146**, e1147–e1159. 10.1016/j.wneu.2020.11.113 (2021). 33259973 10.1016/j.wneu.2020.11.113

[27] AlKubeyyer, A., Ben Ismail, M. M., Bchir, O. & Alkubeyyer, M. Automatic detection of the meningioma tumour firmness in MRI images. *J. Xray Sci. Technol.* **28**, 659–682. 10.3233/XST-200644 (2020). 32538892 10.3233/XST-200644

[28] Zhang, J. *et al.* Radiomic features of magnetic resonance images as novel preoperative predictive factors of bone invasion in meningiomas. *Eur J Radiol* **132**, 109287. 10.1016/j.ejrad.2020.109287 (2020). 32980725 10.1016/j.ejrad.2020.109287

[29] Zhang, J. *et al.* A radiomics model for preoperative prediction of brain invasion in meningioma non-invasively based on MRI: A multicentre study. *EBioMedicine* **58**, 102933. 10.1016/j.ebiom.2020.102933 (2020). 32739863 10.1016/j.ebiom.2020.102933 PMC7393568

[30] Joo, L. *et al.* Extensive peritumoural edema and brain-to-tumour interface MRI features enable prediction of brain invasion in meningioma: development and validation. *Neuro Oncol.* **23**, 324–333. 10.1093/neuonc/noaa190 (2021). 32789495 10.1093/neuonc/noaa190 PMC8631067

[31] Florez, E. *et al.* Multiparametric magnetic resonance imaging in the assessment of primary brain tumours through radiomic features: A metric for guided radiation treatment planning. *Cureus* **10**, e3426. 10.7759/cureus.3426 (2018). 30542636 10.7759/cureus.3426 PMC6284876

[32] Kandemirli, S. G. *et al.* Presurgical detection of brain invasion status in meningiomas based on first-order histogram based texture analysis of contrast enhanced imaging. *Clin. Neurol. Neurosurg.* **198**, 106205. 10.1016/j.clineuro.2020.106205 (2020). 32932028 10.1016/j.clineuro.2020.106205

[33] Baumgarten, P. *et al.* Brain invasion in otherwise benign meningiomas does not predict tumour recurrence. *Acta Neuropathol.* **132**, 479–481. 10.1007/s00401-016-1598-1 (2016). 27464983 10.1007/s00401-016-1598-1

[34] Behling, F., Hempel, J. M. & Schittenhelm, J. Brain invasion in meningioma—A prognostic potential worth exploring. *Cancers (Basel).*10.3390/cancers13133259 (2021). 34209798 10.3390/cancers13133259 PMC8267840

[35] Li, N. *et al.* A clinical semantic and radiomics nomogram for predicting brain invasion in WHO grade II meningioma based on tumour and tumour-to-brain interface features. *Front. Oncol.* **11**, 752158. 10.3389/fonc.2021.752158 (2021). 34745982 10.3389/fonc.2021.752158 PMC8570084

[36] He, L. *et al.* Effects of contrast-enhancement, reconstruction slice thickness and convolution kernel on the diagnostic performance of radiomics signature in solitary pulmonary nodule. *Sci. Rep.* **6**, 34921. 10.1038/srep34921 (2016). 27721474 10.1038/srep34921 PMC5056507

[37] Avery, E., Sanelli, P. C., Aboian, M. & Payabvash, S. Radiomics: A primer on processing workflow and analysis. *Semin. Ultrasound CT MR* **43**, 142–146. 10.1053/j.sult.2022.02.003 (2022). 35339254 10.1053/j.sult.2022.02.003 PMC8961004

[38] Clarke, R. *et al.* The properties of high-dimensional data spaces: Implications for exploring gene and protein expression data. *Nat. Rev. Cancer* **8**, 37–49. 10.1038/nrc2294 (2008). 18097463 10.1038/nrc2294 PMC2238676

[39] Ferte, C. *et al.* Impact of bioinformatic procedures in the development and translation of high-throughput molecular classifiers in oncology. *Clin. Cancer Res.* **19**, 4315–4325. 10.1158/1078-0432.CCR-12-3937 (2013). 23780890 10.1158/1078-0432.CCR-12-3937 PMC3745509

[40] Yao, J., Mao, Q., Goodison, S., Mai, V. & Sun, Y. Feature selection for unsupervised learning through local learning. *Pattern Recognit. Lett.* **53**, 100–107. 10.1016/j.patrec.2014.11.006 (2015). 10.1016/j.patrec.2014.11.006

