# Supplementary material for: Preoperative prediction of CNS WHO grade and tumour aggressiveness in intracranial meningioma based on radiomics and structured semantics
Source: Sci Rep. 2024 Sep 4;14:20586. doi: 10.1038/s41598-024-71200-0 (PMC11374997; doi:10.1038/s41598-024-71200-0)
Supplement: Supplementary file 1 — Supplementary Figure S1. [file 41598_2024_71200_MOESM1_ESM.docx]

**Supplementary Information for**

**Preoperative prediction of CNS WHO grade and tumour aggressiveness in intracranial meningioma based on radiomics and structured semantics**

Darius Kalasauskas^1^*, Michael Kosterhon^1^*, Elena Kurz^1^, Leon Schmidt^1^, Sebastian Altmann^2^, Nils F. Grauhan^2^, Clemens Sommer^3^, Ahmed Othman^2^, Marc A. Brockmann^2^, Florian Ringel^1^, Naureen Keric^1^

1 Department of Neurosurgery, 2 Department of Neuroradiology, 3 Institute of Neuropathology, University Medical Center, Johannes Gutenberg University, Mainz, Germany

*The authors contributed equally and share first authorship

Corresponding author:

Michael Kosterhon (ORCID: 0000-0002-8811-4699)

Department of Neurosurgery

University Medical Center, Johannes Gutenberg University,

Langenbeckstr. 1

55131 Mainz, Germany

[mikoster@uni-mainz.de](mailto:mikoster@uni-mainz.de)


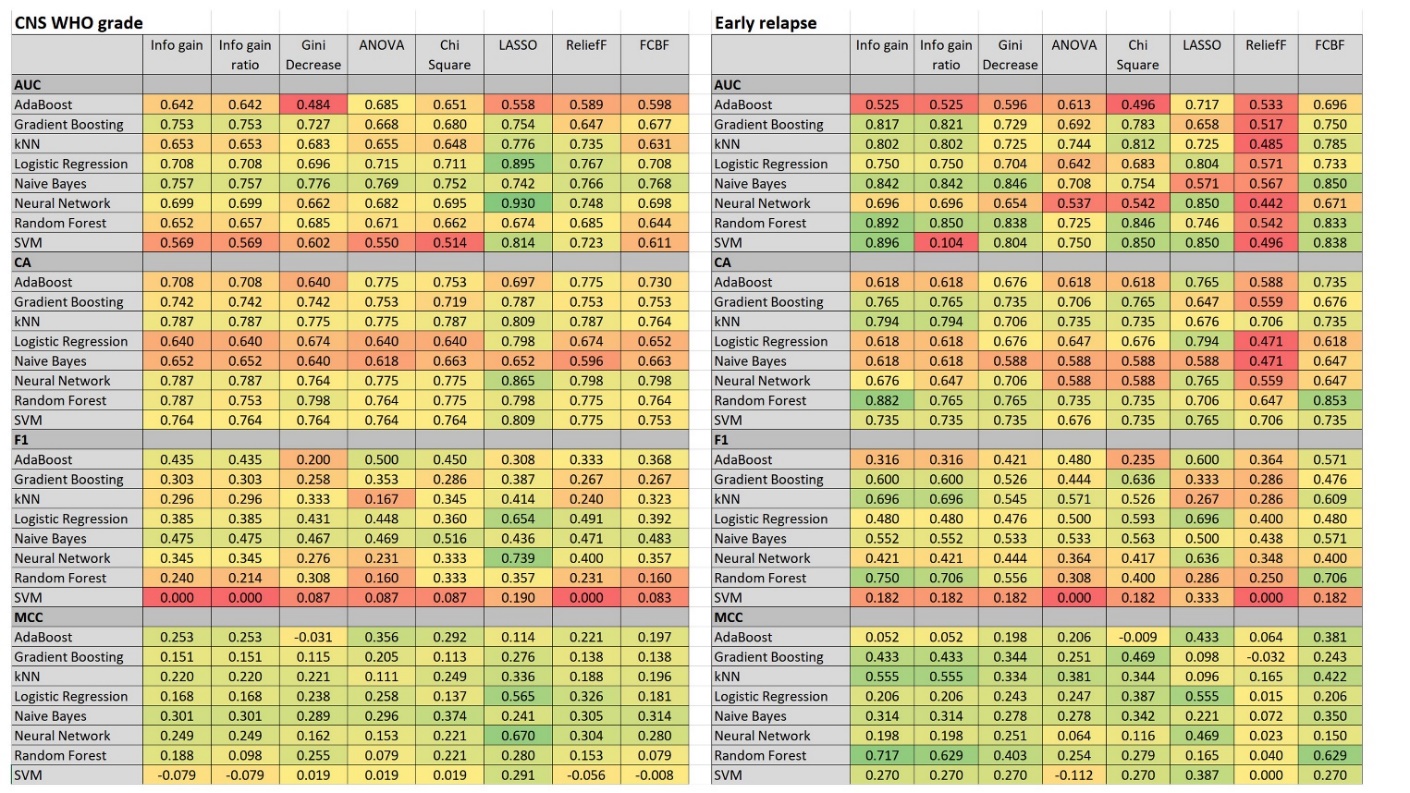


**Supplementary Fig. 1** AUC under the ROC, Classification Accuracy (CA), F1-Score and Matthews correlation coefficient (MCC) for different combinations of radiomics feature selection methods (colums) and different machine learning classifiers (rows) for detection of CNS WHO grade II (left table) and detection of early tumour recurrence (right table).
